# The Effects of Chinese Parenting Belief on Preschoolers’ Temperament and Secure Attachment

**DOI:** 10.3390/children10010009

**Published:** 2022-12-21

**Authors:** Chung Chin Wu

**Affiliations:** Department of Early Childhood Education, National Pingtung University, Pingtung 900391, Taiwan; minin72704@mail.nptu.edu.tw

**Keywords:** Chinese, parenting belief, preschoolers’ temperament, secure attachment

## Abstract

In the past, there were a few studies investigating the effects of parenting belief on preschoolers’ temperament and secure attachment. There were some inconsistencies; some effects were also unclear. A total of 2164 parents of three-year-old preschoolers were selected for a panel study named National Longitudinal Study of Child Development and Care. At first, confirmatory factor analysis was conducted to examine construct validity of Chinese parenting belief, preschoolers’ temperament, and secure attachment. Consecutively, the effects of Chinese parenting belief on preschoolers’ temperament and secure attachment were investigated through structural equation modeling. Results showed: (1) construct validity of Chinese parenting belief (composed of Guan, Jiao, achievement, and Chi beliefs), preschoolers’ temperament (composed of extraversion, effortful control, and negative affection temperament) and secure attachment were good. (2) The Guan belief showed no effects on all temperaments and secure attachment. (3) Only Jiao belief contributed to the development of extraversion, effortful control, negative affection, and secure attachment. (4) The Achievement belief had a detrimental effect on the development of secure attachment, but it had no effects on others. (5) The Chi belief can increase the development of negative affection temperament, but it had no effects on the rest.

## 1. Introduction

The effects of parenting style on several outcomes of children (e.g., achievement) is a widely studied topic [1,2]. In contrast, research focusing on parenting beliefs, a moderator between the relationships between parenting style and outcomes, have received relatively less attention. The effects of parenting style may be also vary from culture to culture [3,4]. It means that whether parenting style may take effect is conditioned simultaneously on parenting beliefs and culture. In this regard, the effects of Chinese parenting beliefs on several outcomes (e.g., temperament and attachment) were less clear, and it may deserve more discussions.

Parenting beliefs refer to the intentions, expectations, and values toward children while parents are rearing and educating their children. Four Chinese parenting beliefs are salient in Chinese culture. They are the discipline belief (namely “*Guan*”), the guidance belief (namely “*Jiao*”), the *achievement* belief, and the shame belief (namely “*Chi*”). The *Guan* belief refers to parents’ belief that control is necessary for cultivating children’s positive behaviors [5,6,7,8]. Chinese parents gave more punishments, reprimands, admonishments, and emphasized absolute obedience [9,10,11,12]. It has been demonstrated that guan will significantly increase children’s anti-social behaviors [13]. The *Jiao* belief refers to parents tend to adopt autonomous and supportive guidance or instruction to support children’s positive development [14,15,16]. It was found that this belief may be beneficial for children to perform behaviors corresponding to parents’ expectations [17]. The *achievement* belief refers to parents’ expecting or even demanding that their children get better grades or perform better in school to honor them or their family [18,19]. The Chi belief refers to parents usually reminding their children they will feel shameful for their children’s inappropriate behaviors [20,21,22], and it was considered to have more negative effects on children’s psychological wellbeing [23].

Researchers demonstrated three types of temperament for preschoolers; they are extraversion, effortful control, and negative affection, respectively [24,25]. Extraversion temperament reflects the preferred amount of physical activity (e.g., prefer dynamic activities more than static activities), reaction intensity (e.g., actively engage in new activities), and the tendency of approach and avoidance of children (e.g., more like to play with different children). Effortful control temperament reflects children’s adaptability, distraction, the span of attention, and persistence in a certain activity. Negative affection temperament reflects children’s response threshold and emotionality (e.g., subject to anger to others’ verbal provocation). Parenting beliefs are influential factors for temperament [26].

By adopting the framework of parenting style and incorporating Western samples, there was found a positive relationship between authoritarian belief and negative affection temperament [27,28]. Similarly, it was also indicated that punitive parenting increases negative affectivity [29]. Another study further found that authoritative belief may positively predict infants’ extraversion temperament [30,31] and effortful control temperament [28,30]. It was found that both authoritative belief and permissive belief positively predicted effortful control temperament [32,33]. There were some evidence that indicated that authoritative belief has no effect on negative affectivity [34,35]. By adopting the same framework of parenting style but incorporating Chinese or Eastern samples, it was found that there was a positive relationship between the authoritative parenting of Chinese parents and the extraversion temperament, but authoritarian parenting of Chinese parents was negatively related to extraversion temperament [36] and positively predicted effortful control temperament [37]. In another study, the authoritarian parenting of Chinese and Indian parents was found to negatively predict effortful control temperament, and positive predict negative affection temperament [38,39]. However, the authoritative parenting of Chinese parents was unable to predict these two kinds of temperament [38]. However, these effects of parenting on temperament are based on the framework of parenting style which was constructed based on Western culture. It is less clear whether the effects of Chinese parenting beliefs on temperament would be similar to the effects of parenting style.

By adapting another framework closed to Chinese parenting belief, there were some evidences showed that the “*Guan*” belief may promote children to develop extraversion temperament, effortful control temperament and negative affection temperament [40]. However, it was also found that the *Guan* belief of the Chinese mother was unrelated to [41] or negatively related to negative affection temperament [42], whereas the *Jiao* belief was demonstrated to reduce negative affection temperament [43], but its effects on extraversion temperament and effortful control temperament were unclear. In addition, the *achievement* belief was negatively related to extraversion temperament, but positive related to effortful control temperament [44]. On the contrary, some researchers found that parental educational involvement is protective factor in academic adjustment (e.g., the feeling of belonging in the educational environment and prosocial behaviors) [45]. It suggested that the *achievement* belief may be beneficial for promoting the development of adaptive temperaments (i.e., extraversion temperament and effortful control temperament) and for inhibiting the development of maladaptive temperament (i.e., negative affection temperament). However, its direct effects of the *“achievement*” belief on negative affection temperament were also far from clear. Finally, the *Chi* belief was unrelated to extraversion temperament [46], and effortful control temperament [37,46], but it was positively related to negative affection temperament [46,47].

Parenting belief is also influential for secure attachment. Attachment play an important role in one’s socio-emotional development, and it refers to the affect connections between preschoolers and main caregivers; secure attachment in the infant stage increasingly formed intensive positive affection to regulate their emotions and behaviors based on the responsive reactions of their caregivers [48,49]. There are three styles of attachment; they are secure, insecure ambivalent, and insecure avoidant attachment, respectively. Secure attachment is developed when parents are responsive to children’s physical or psychological needs. Insecure ambivalent attachment is produced when parents are selectively responded to children’s certain needs regardless of others. Insecure avoidant attachment is occurred when parents have no response to children’s needs. Secure attachment may influence cognition, emotion, life accommodation, and intimate relationships with others in adolescent period [50,51], while the other two attachment styles may lead to several maladaptive outcomes (e.g., higher negative emotionality) [52]. The development of secure attachment may be challenged when young children initially encounter an environmental transition from the family to the preschool setting. It is particularly important to clarify the protective and detrimental effects of parenting belief on the development of secure attachment. Researchers found a positive relationship between authoritative belief and secure attachment [53], and between the “*Guan*” belief and secure attachment [42]. Similarly, a meta-analysis including a Chinese sample also demonstrated that the *Jiao* belief may be beneficial for developing secure attachment [54]. However, some researchers found that both “*Guan*” belief and “*Jiao*” belief were unrelated to secure attachment [55]. Few studies investigate the effect of the “achievement” belief on secure attachment. Inferring indirectly from empirical evidence, it indicates that the lower parents possess achievement belief the more responsiveness they give to their children, and the responsiveness is beneficial to the development of secure attachment. It suggests that achievement belief may be negatively related to secure attachment [18]. Similarly, Xu et al. demonstrated that *Chi* belief has a positive relationship with insecure attachment; it implied that *Chi* belief may also have a negative relationship with secure attachment [22].

Apparently, findings regarding the effects of Chinese parenting beliefs on both temperaments and secure attachment were not only inconclusive, but some were unclear. Further investigations are needed to contribute to our understanding about better Chinese parenting beliefs for cultivating children’s adaptive temperaments and secure attachment. Consequently, the main purposes of present study were twofold:

- To investigate the effects of Chinese parenting beliefs on preschoolers’ temperaments.
- To clarify the effects of Chinese parenting beliefs on preschoolers’ secure attachment.

## 2. Methodology

### 2.1. Participants

Data is retrieved from National Longitudinal Study of Child Development and Care (NLSCDC) database in Taiwan. This panel study was reviewed and approved by the National Taiwan University and National Taiwan Normal University Subjects Institutional Review Boards. One of the main purposes of NLSCDC is to investigate the effects of the family psychological environment on the development of cognition and social emotion for young children aged from birth to age 8. Stratified two-stage probability proportional to size sampling was applied in this panel study. All the 358 administration areas in Taiwan were taken as primary sampling units, and they were integrated to form 19 stratifications: 7 in the northeast, 7 in the middle, 3 in the southeast, and 2 in the west of Taiwan. Individuals were served as the secondary sampling units, and they were selected based on household registration information. There were 1140, 709, 303 and 48 parents paired with their three-year-old children selected from the northeast, the middle, the southeast, and the west of Taiwan, respectively. A total of 2164 representative parents and their three-year-old children (1113 boys and 1051 girls) were included in this study [56]. About 4/5 mothers and 1/5 fathers participated to complete the questionnaires. There were about 1/6 fathers and 1/8 mothers who held a master’s degree. Participants were informed that all of their responses were kept confidential, and their treatments in the preschools would not be influenced by their participation. Three-year-old children were selected because this age is the first year for most of children enroll in informal school settings. They experienced a transition from the family to a totally strange environment, and they have to adapt themselves to various new conditions. Consequently, they may experience considerable emotional disturbances, and their socio-emotional development may be affected to some extent. This study was concerned with investigating how parenting beliefs exert effects on their temperament and secure attachment.

### 2.2. Instruments

There were three instruments used in this study. They were developed by and were used in NLSCDC to understand the effects of family psychological environment on socio-emotional development as one of the main purposes of this panel study, which corresponded to the main purposes of this study. All instruments were validated by reliability analysis, factor analysis, and item response analysis [56].

#### 2.2.1. Chinese Parenting Belief Scale

There are nine items in the Chinese parenting belief scale; it is composed of four subscales: the *Guan* belief, *Jiao* belief, *Achievement* belief, and *Chi* belief subscales, respectively. There are three items for measuring *Guan* belief. A sample item is: chide preschooler will let them progress. There are two items for measuring *Jiao* belief; a sample item is: Preschooler should look at me when I am talking with him/her. There are two items for measuring *Achievement* belief; a sample item is: I feel sense of achievement once they perform better on leaning. Two items are designed to measure *Chi* belief; a sample item is: I should feel shame if preschooler is unruly outside. A four-point Likert’s scale design was used, and parents were required to choose one from the four options on a 1 (“very disagree”) to 4 (“very disagree”) scale. The internal consistency coefficients for the four subscales are 0.70, 0.72, 0.73 and 0.74, respectively.

#### 2.2.2. Preschooler Temperament Scale

There are 11 items in the preschooler temperament scale; it is composed of three subscales: extraversion temperament, effortful control temperament, and negative affection temperament subscales, respectively. There are three items for measuring extraversion temperament. A sample item is: Comparing to static activities, preschooler is more like dynamic activities. There are four items for measuring effortful control temperament; a sample item is: Preschooler consistently engaged in an activity or played a toy for a long time. There are four items for measuring negative affection temperament; a sample item is: Preschooler get angry easily. A five-point Likert’s scale design was used, and parents were required to choose one from the five options on a 1 (“never”) to 5 (“always”) scale. The internal consistency coefficients for three subscales are 0.66, 0.63 and 0.60, respectively.

#### 2.2.3. Preschooler Secure Attachment Scale

There are four items in the preschooler secure attachment scale; a sample item is: Preschooler will look for me when I am not staying with him/her. A five-point Likert’s scale design was used; parents were required to choose one from the five options on a 1 (“never”) to 5 (“always”) scale. Preschoolers who score lower on this scale are highly securely attached to their parents. The internal consistency coefficient is 0.66.

### 2.3. Analysis

Mplus 8.0 is used to analyze the measurement model and the structural model by using structural equation modeling (SEM). Several goodness of fit indices were used to evaluate model fit. They are χ^2^, RMSEA (root mean square error of approximation), CFI (comparative fit index), TLI (Tucker–Lewis index), and SRMR (standardized root mean square residual). Non-significant χ^2^ values indicated model fit to the data, but it is easily affect by a larger sample size. Therefore, other indices are mainly used to evaluate model fit. RMSEA ≤ 0.06, and CFI and TLI ≥ 0.95 was considered a model that fit the data well. A value of 0.06 < RMSEA ≤ 0.08, and CFI and TLI ≥ 0.90 was considered a model fit that was acceptable [57,58]. Confirmatory factor analyses (CFAs) for three constructs were conducted in advance. CFAs were conducted by using MLR (Maximum likelihood with robust standard errors). After that, SEM was implemented to investigate the structural relationships among latent variables. In the hypothesized SEM model, there are four exogenous latent variables (*Guan* belief, *Jiao* belief, *Achievement* belief and *Chi* belief) and four endogenous latent variables (extraversion temperament, effortful control temperament, negative affection temperament and secure attachment). The effects of Chinese parenting beliefs on preschoolers’ temperament and secure attachment were investigated in this model.

## 3. Results

### 3.1. Preliminary Analysis

Item means of Chinese parenting beliefs ranged from 2.40 to 3.23 (SD ranged from 0.56 to 0.76; correlation coefficients ranged from −0.05 to 0.57). Means of items for preschooler’s temperament ranged from 2.76 to 4.29 (SD ranged from 0.82 to 1.32; correlation coefficients ranged from −0.04 to 0.43). Means of items for preschooler’s secure attachment ranged from 3.49 to 4.62 (SD ranged from 0.66 to 1.29; correlation coefficients ranged from 0.18 to 0.31).

### 3.2. The Goodness of Fit of Measurement Model

The goodness of fit of measurement model was evaluated to assure that items correctly loaded on their latent constructs before investigating the effects of Chinese parenting beliefs on temperament and secure attachment. The measurement model for Chinese parenting belief was composed of four latent variables. CFA results for this model showed that each of the fit indices met the criteria for a good-fitting model: χ^2^(21, N = 2164) = 127.59, *p* < 0.05, CFI = 0.97, TLI = 0.95, RMSEA = 0.048 (90% CI ranged from 0.041 to 0.057). Intercorrelation coefficients among these four latent variables ranged from 0.26 to 0.64 (95% CI ranged from 0.20 to 0.70, 1 is not included in this range). CFA results for the measurement model of Chinese parenting beliefs demonstrated a good construct validity and latent variables in this model were distinguishable. Consequently, this measurement model could be further used to investigate their effects to temperament and secure attachment.

Measurement model for preschooler’s temperament composed of three latent variables. CFA results for this model showed that each of the fit indices generally met the criteria for an acceptable model: χ^2^(2, N = 2164) = 235.25, *p* < 0.05, CFI = 0.93, TLI = 0.91, RMSEA = 0.047 (90%CI ranged from 0.041 to 0.053). Intercorrelation coefficients among these four latent variables ranged from 0.12 to 0.51 (95% CI ranged from 0.04 to 0.58, 1 is not included in this range). CFA results for the measurement model for preschooler’s secure attachment also met the criteria for a good-fitting model: χ^2^(41, N = 2164) = 22.25, *p* < 0.05, CFI = 0.96, TLI = 0.90, RMSEA = 0.068 (90% CI ranged from 0.045 to 0.095). Correlations among all latent variables ranged from 0.12 to 0.64 (*p*s ranged from 0.00 to 0.02 < 0.05).

In general, CFA results for these two measurement models of preschooler’s temperament and secure attachment also demonstrated good construct validities, and latent variables in the measurement model of temperament were distinguishable. Consequently, these measurement models could be used in further analysis.

**Figure 1:**
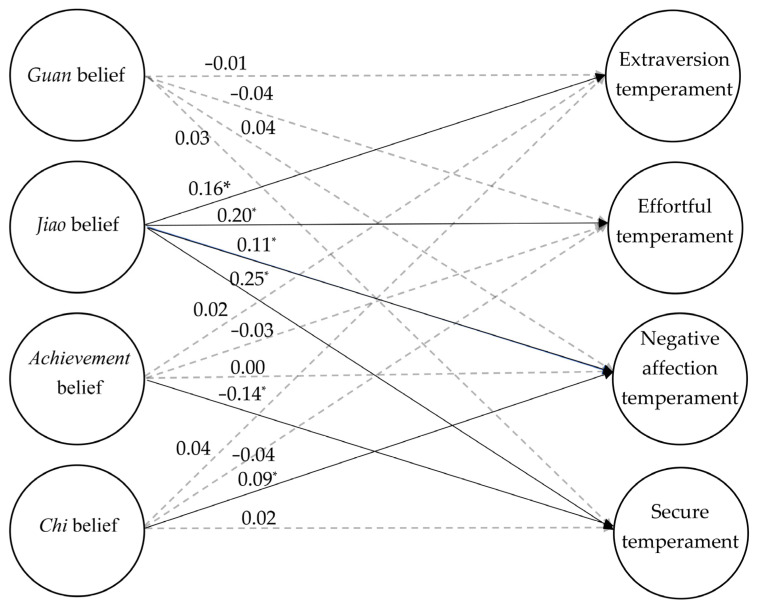
The graphical representation of the effects of Chinese parenting beliefs on temperament and secure attachment. * *p* < 0.05.

The overall structural model, including three measurement models for each latent variable, generally met the criteria for a good-fitting model: χ^2^(224, N = 2164) = 834.03, *p* < 0.05, CFI = 0.93, TLI = 0.91, RMSEA = 0.035 (90% CI ranged from 0.033 to 0.038). It suggested that the effects of Chinese parenting beliefs on temperament and secure attachment could be investigated based on this good fitted structural model. Table 1 shows the effects of Chinese parenting beliefs on temperament and secure attachment (Figure 1 shows the graphical representation).

#### 3.2.1. The Effects of “Guan” Belief on Temperament and Secure Attachment

As it can be seen from Table 1, *Guan* belief shows no effects on extraversion temperament, effortful control temperament, negative affection temperament, and secure attachment (β_1_, β_2_, β_3_, and β_4_ are −0.01, −0.04, 0.04 and 0.03, respectively, *p*s = 0.91, 0.43, 0.43, and 0.56 > 0.05).

#### 3.2.2. The Effects of “Jiao” Belief on Temperament and Secure Attachment

In contrast to above findings, *Jiao* belief shows positive effects on extraversion temperament, effortful control temperament, negative affection temperament, and secure attachment (β_5_, β_6_, β_7_ and β_8_ are 0.16, 0.20, 0.11 and 0.25, respectively, *p*s = 0.00, 0.00, 0.01 and 0.00 < 0.05).

#### 3.2.3. The Effects of “Achievement” Belief on Temperament and Secure Attachment

*Achievement* belief shows no effects on extraversion temperament, effortful control temperament, and negative affection temperament (β_9_, β_10_, and β_11_ are 0.02, −0.03, and 0.00, respectively, *p*s = 0.76, 0.68 and 0.95 > 0.05). However, it has a negative effect on secure attachment (β_12_ is −0.14, *p* = 0.03 < 0.05).

#### 3.2.4. The Effects of “Chi” Belief on Temperament and Secure Attachment

*Chi* belief shows no effects on extraversion temperament, effortful control temperament, and secure attachment (β_13_, β_14_, and β_16_ are 0.04, −0.04, and 0.02, respectively, *p*s = 0.43, 0.36 and 0.75 > 0.05). However, it has positive effect on negative affection temperament (β_15_ is 0.09, *p* = 0.04 < 0.05).

## 4. Discussion and Conclusion

It has been argued that parenting has considerably differences between Western and Eastern country [59], and Chinese parenting belief cannot be fully understood by the theoretical framework of parenting style which was developed based on Western samples in the Western countries [60]. Chinese parenting beliefs were constructed according to related researches [5,6,14,15,18,19,20,21,22,41]. Results of CFAs are clearly showed that Chinese parenting belief are composed of four dimensions as expectation, they are *Guan* belief, *Jiao* belief, *Achievement* belief, and *Chi* belief, respectively. Moreover, they are different constructs which may reflect different social meanings in Chinese society. It indicated that the construed Chinese parenting beliefs was supported by present study. In addition, although temperament construct is proposed grounded in Western culture, its contents are also supported by Chinese preschooler sample.

The *Guan* belief was found to have no effects on preschooler’s temperaments and secure attachment. These findings are inconsistent with those of the researchers who adapted parenting type as a research framework. Specifically, the meaning of the *Guan* belief is closed to authoritarian belief, but it showed neither a positive relationship with negative affection temperament [25,30], nor a negative relationship with extraversion temperament [36], or effortful control temperament [38]. In contrast, these findings are very similar to those studies that adapted Chinese parenting beliefs as a research framework and incorporated Chinese parents as samples. Specifically, researchers found that the *Guan* belief was unrelated to negative affection temperament and secure attachment [41,55]. Present findings showed that there are two null effects of the *Guan* belief on extraversion temperament and effortful control temperament, which expand our current understanding and use it to encourage parents to discard this parenting belief when they are educating and caring for their children. In addition, these results only implied that there were no temporary effects of the *Guan* belief on temperament and secure attachment. However, traditionally, the guan belief implied that parents knew the importance of punishments, reprimands, and admonishment for cultivating the conformity to society. It may direct children to fear of engaging in social activities to avoid doing something wrong, to persist in learning activities that are demanding, and to inhibit their negative affections [5]. Moreover, harsh discipline may be also detrimental for cultivating secure attachment. Consequently, in the long-term, it was unclear whether it contributes to the formation of effortful control temperament and has negative effects on extraversion, negative affection temperament, and secure attachment.

In contrast to the effects of the *Guan* belief, the *Jiao* belief was found to have positive effects on preschooler’s temperaments and secure attachment. These finding were relatively consistent with former researches [30,32,33,36,53,54]. However, the positive effect of the *Jiao* belief on negative affection temperament was unexpected, and it contradicted former research findings which indicated that the *Jiao* belief may contribute to reduce the influences of negative affection temperament [43]. It may be implied that there are moderators (e.g., environmental transition) between the *Jiao* belief and negative affection temperament. For example, 3-year-old preschoolers had just started to enroll in kindergarten in Taiwan, and they were unfamiliar to this new learning environment. They may produce emotional fluctuations when they situate in this strange environment, and this context may exert considerable positive effects on negative affection temperament. Consequently, the expected negative effect of the *Jiao* belief on negative affection temperament was overturned by the possibly positive contextual effect. These findings contributed to make conclusions on the positive effects of the *Jiao* belief on extraversion temperament, effortful control temperament, and secure attachment. In addition, it also suggested further studies were needed to clarify the effect on negative affection temperament.

The *achievement* belief was found to have no effects on extraversion temperament, effortful control temperament, and negative affection temperament. It contradicted former findings that indicated its negative relationship with extraversion temperament and its positive relationship with effortful control temperament [44]. In Chinese society, parents may long to see their children become successful and have a bright future to get superior social status to make their parents feel proud or honor their clan [61], and this belief may direct children to focus on cognitive performances by using various motivational strategies to approach success (e.g., persistence on a learning task to complete it). It is expected that the *achievement* belief may contribute to the formation of effortful control temperament, but this effect was not found. It may be implied that achievement belief may need more time to exert its positive effect on effortful control temperament, or it may be also suggested that this effect is significant in the achievement context (e.g., formal schooling) involving more social comparison or competition. Moreover, it is reasonable that there are no effects of achievement belief on extraversion and negative affection temperament because the *achievement* belief is only expected to direct children’s cognitive performances rather than social or affection domain. The negative effect of the *achievement* belief on secure attachment was consistent with the hypothesis which was inferred from Chen and Chou’s finding [18]. These findings simultaneously contributed to expand our understanding of the unclear relationship between the *achievement* belief and negative affection temperament, to provide information on the negative effect of achievement on secure attachment, and to encourage future researchers to investigate the contradictory results.

Finally, the *Chi* belief was found to have no effects on extraversion temperament, effortful control temperament, and secure attachment. It was quite consistent with former researchers who found that the *Chi* belief was unrelated to extraversion temperament and effortful control temperament [46], but it was positively related to negative affection temperament [46,47]. Chinese parents were heavily influenced by Confucian traditions; it suggested that Chinese parents taught their children to conduct themselves so as to not disgrace to their parents and clan. It makes children sensitive to social norms and feel shame when they fail to obey these rules [37]. Theoretically, the *Chi* parenting may guide parents to demand their children to adapt to social rules, and in turn to cultivate effortful control temperament. Unexpectedly, the *Chi* belief has no effect on effortful control temperament. It implied that this effect may take time to be revealed. In addition, it has been demonstrated that there were emotional contagions among people [62], and the positive effect of the *Chi* belief on negative affection temperament suggested that Chinese parents may transmit negative emotional cues to their children unconsciously. Consequently, this belief imperceptibly fosters children’s negative affection temperament. Furthermore, it was found that one of the results is not consistent with the hypothesis which inferred from Xu et al.’s study, suggesting the *Chi* belief may exert a negative effect on secure attachment [22]. These findings may contribute to understand the effects of the *Chi* belief on extraversion temperament, effortful control temperament, and negative affection temperament. It will also help future studies to further clarify the effects of *Chi* on secure attachment.

In general, practically, the *Jiao* belief may be encouraged with caution because it was not clear whether it may contribute to promoting negative affection temperament. In contrast, parents’ *achievement* belief and *Chi* belief may be discouraged to prevent their potential negative effects on adaptive temperament (e.g., effortful control temperament) and/or secure attachment, and to promote possible positive effects on maladaptive temperament (e.g., negative affection temperament) and/or secure attachment.

## 5. Limitations

Present findings may reflect short-term phenomena due to the nature of the cross-sectional study. It was unclear whether these effects may disappear or reveal in any expected or unexpected directions as time goes on. For example, the null effects of the ***Guan*** belief on negative affection temperament may be temporary and may be turned into negative in the future. More studies were clearly needed to focus on the longitudinal effects of Chinese parenting beliefs on the development of temperaments and secure attachment. In addition, some interest findings (e.g., the positive effect of the ***Jiao*** belief on negative affection temperament) may be conditioned on other factors (e.g., the contextual transition) which was not expected and included in this study. These potential factors should be considered in future studies to further clarify present findings.

## Figures and Tables

**Table 1:** The structural coefficients of Chinese parenting beliefs on preschooler’s temperament and secure attachment.

Structural Paths	Coefficients
“*Guan*” belief→Extraversion temperament (*β*_1_)	−0.01
“*Guan*” belief→Effortful control temperament (*β*_2_)	−0.04
“*Guan*” belief→Negative affection temperament(*β*_3_)	0.04
“*Guan*” belief→Secure attachment (*β*_4_)	0.03
“*Jiao*” belief→Extraversion temperament (*β*_5_)	0.16 *
“*Jiao*” belief→Effortful control temperament (*β*_6_)	0.20 *
“*Jiao*” belief→Negative affection temperament (*β*_7_)	0.11 *
“*Jiao*” belief→Secure attachment (*β*_8_)	0.25 *
“*Achievement*” belief→Extraversion temperament (*β*_9_)	0.02
“*Achievement*” belief→Effortful control temperament (*β*_10_)	−0.03
“*Achievement*” belief→Negative affection temperament (*β*_11_)	0.00
“*Achievement*” belief→Secure attachment (*β*_12_)	−0.14 *
“*Chi*” belief→Extraversion temperament (*β*_13_)	0.04
“*Chi*” belief→Effortful control temperament (*β*_14_)	−0.04
“*Chi*” belief→Negative affection temperament(*β*_15_)	0.09 *
“*Chi*” belief→Secure attachment (*β*_16_)	0.02

Note: Coefficients are completely standardized coefficients. * *p* < 0.05.

## Data Availability

This article used publicly available data with no protected health information.
